# Supplementary material for: A rapid approach for sex assignment by RAD-seq using a reference genome
Source: PLoS One. 2024 Apr 5;19(4):e0297987. doi: 10.1371/journal.pone.0297987 (PMC10997085; doi:10.1371/journal.pone.0297987)
Supplement: S1 File — It will be also available on protocols.io. (PDF) [file pone.0297987.s001.pdf]

C2WKYFCW

Protocol Info: Diego Peralta, Juan I. Túnez, Ulises E. Rodríguez Cruz, Santiago G. Ceballos . A rapid approach for sex assignment by RAD-seq using a reference genome. **protocols.io** https://protocols.io/view/a-rapid-approach-for-sex-assignment-by-rad-seq-usi-c2wkyfcwVersion created by Diego Peralta

Created: Oct 03, 2023

Last Modified: Oct 03, 2023

PROTOCOL integer ID: 88748

A rapid approach for sex assignment by RAD-seq using a reference genome V.(c2wkyfcw)

Diego Peralta[1,2], Juan I. Túnez[1,3], Ulises E. Rodríguez Cruz[4], Santiago G. Ceballos[5,6]

[1]Grupo de Investigación en Ecología Molecular, Instituto de Ecología y Desarrollo Sustentable (INEDES-CONICET-UNLu-CIC), Luján, Argentina;
[2]Departamento de Ecología de la Diversidad, Instituto de Ecología, Universidad Nacional Autónoma de México, Ciudad de México, México;
[3]Departamento de Ciencias Básicas, Universidad Nacional de Luján, Luján, Argentina;
[4]Departamento de Ecología Evolutiva, Instituto de Ecología, Universidad Nacional Autónoma de México, Ciudad de México, México;
[5]Instituto de Ciencias Polares, Ambiente y Recursos Naturales, Universidad Nacional de Tierra del Fuego, Ushuaia, Argentina;
[6]Centro Austral de Investigaciones Científicas (CADIC-CONICET), Ushuaia, Argentina

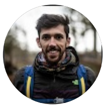

Diego Peralta

ABSTRACT

Sex identification is a common objective in molecular ecology. While many vertebrates display sexual dimorphism, determining the sex can be challenging in certain situations, such as species lacking clear sex-related phenotypic characteristics or in studies using non-invasive methods. In these cases, DNA analyses serve as valuable tools not only for sex determination but also for validating sex assignment based on phenotypic traits. In this study, we developed a bioinformatic framework for sex assignment using genomic data obtained through GBS, and having an available closely related genome assembled at the chromosome level. Our method consists of two ad hoc indexes that rely on the different properties of the mammalian heteromorphic sex chromosomes. For this purpose, we mapped RAD-seq loci to a reference genome and then obtained missingness and coverage depth values for the autosomes and X and Y chromosomes of each individual. Our methodology successfully determined the sex of 165 fur seals that had been phenotypically sexed in a previous study and 40 sea lions sampled in a non-invasive way. Additionally, we evaluated the accuracy of each index in sequences with varying average coverage depths, with Index Y proving greater reliability and robustness in assigning sex to individuals with low-depth coverage. We believe that the approach presented here can be extended to any animal taxa with known heteromorphic XY/ZW sex chromosome systems and that it can tolerate various qualities of GBS sequencing data.

1

Diego M. Peralta, Juan I. Túnez, Ulises E. Rodríguez Cruz, Santiago G. Ceballos

2 Objective

Determining the sex of different individuals using GBS and a reference genome, based on the distinct properties of mammalian sex chromosomes X and Y.

3 Requirements

The requirements are listed according to their utilization.

- Linux
- BWA (0.7.17-r1188)
- Samtools (1.3.1)
- Stacks (2.64)
- VCFtools (0.1.17)
- R (4.3.0)

Versions may differ from those used here.

4 Usage

Here is a detailed step-by-step procedure, beginning with raw sequences and concluding with sex identification.

Alingment to a Reference Genome

1.Create a directory, here we call it sexing. Then create the follow subdirectories.

```
sexing/
  files
  bwa
  alignments.bwa
  gstacks.bwa
  populations
```

2.Prepare a tab-delimited population file, popfile.tsv also in the files/ directory. This file describes the populations to which the samples belong.

```
"individual1"TAB"population1"
"individual2"TAB"population2"
```

In our case the file looks like:

```
CB02	1
Q06 1
Q06B 1
PQ03 1
```

BWA

3.Create a Reference Genome Index. Download the reference genome file of the most related organism to your species and put it in files directory.

```
bwa index -p bwa/gac files/genome.fasta > bwa/bwa_index.oe
```

| Single end

```
bwa mem -M -t 16 bwa/gac location_demultiplex/individual1.fq.gz |
samtools view -b > ./alignments.bwa/sample1.bam
```

| Paired end

```
bwa mem -M -t 16 bwa/gac location_demultiplex/individual1.R1.fq.gz
location_demultiplex/individual1.R2.fq.gz | samtools view -b >
./alignments.bwa/sample1.bam
```

| And, next

```
samtools sort -o alignments.bwa/sample1.bam
alignments.bwa/sample1.bam
```

3.1.To avoid making the previous steps for each sample, we appealed to a loop. This must be running from the directory where demultiplex files are stored. *nohup* is an option that allows running programs in the background.

| Single end

```
nohup sh -c
'while read K;
 do
  bwa mem -M -t 16 ../sexing/bwa/gac $K.1.fq.gz | samtools view -b >
../sexing/alignments.bwa/$K.bam;
 done < names.list.txt'
&
```

| Paired end

```
nohup sh -c
'while read K;
 do
  bwa mem -M -t 16 ../sexing/bwa/gac $K.1.fq.gz $K.2.fq.gz |
samtools view -b > ../sexing/alignments.bwa/$K.bam;
 done < names.list.txt'
&
```

| The file “names.list.txt” lists the names used in the analysis sequences of all the individuals, as follows.

```
individual1
individual2
individual3
```

3.2.After this, we sorted the files for each individual in a single step.

```
nohup sh -c
'for K in *.bam;
 do
  samtools sort -o $K $K;
 done'
&
```

gstacks

4.Align reads to reference genome. gstacks module will examine a RAD dataset one locus at a time, looking at all individuals in the metapopulation for that locus.

```
gstacks -I alignments.bwa/ -M files/popfile.tsv -t 24 -O
gstacks.bwa/
```

SNPs Identification

1. 5.Create a catalog of SNPs with populations module. The SNPs filtering steps were aimed to retain as many markers associated with sex chromosomes as possible. Thus, considering that Y linked loci are present only in males, we set the minimum proportion of individuals across populations to process a locus (-R) to 0.3, which assumes a minimum of 30 % of males in our data set. The flag --vcf-all retrieves all the positions within the RAD-loci, containing fixed and variable sites.

```
populations -P gstacks.bwa --popmap files/popfile.tsv --max-obs-het
0.7 -R 0.3 -t 8 -O populations --vcf-all
```

Sex identification

1. 6.Enter to the "populations" directory and obtain files of missingness and depth of coverage of each individual for chromosomes X, Y and the autosomal. VCFtools --chr flag uses loci contained in a specified chromosome, while --not-chr avoids those we chose. Here, we attempted to automate the process. First, create a list of the names of sex chromosomes. To do this, enter to the chosen species genome directory from https://www.ncbi.nlm.nih.gov/genome/ and copy the chromosome identifiers from the column "RefSeq". In our case: Y (NC_045613.1) and X (NC_045612.1). After that, paste them into a new file we call chromosome.list.txt.

```
nano chromosome.list.txt
```

Our file looks like:

```
NC_045613.1
NC_045612.1
```

After saving, make a directory to deposit the new files (we call it “sexing”) and make the next loop to create the files of interest in one step.

```
cd sexing

cat ../chromosome.list.txt | while read line;
 do
  vcftools --chr $line --vcf ../populations.all.vcf --depth --out
${line}

  vcftools --chr $line --vcf ../populations.all.vcf --missing-indv -
-out ${line}
 done

vcftools --not-chr NC_045613.1 --not-chr NC_045612.1 --vcf
../populations.all.vcf --depth --out autosomals

rename 's/$/.tsv/' *.i*
```

Simplify the tables leaving only the first and last columns (sample names and data of interest).

```
for file in *.tsv;
 do
  awk '{print $1, $NF }' $file > ${file/.tsv/_2_.tsv};
 done
```

And named the columns with the name of the chromosome identifiers.

```
for f in *.imiss_2_.tsv;
 do
  n=${f%%.imiss_2_.tsv}
  filename=`echo ${f:r}`; sed -i -e "s/F_MISS/F_MISS_$n/" $f;
 done
```

```
for f in *.idepth_2_.tsv;
 do
  n=${f%%.idepth_2_.tsv}
  filename=`echo ${f:r}`; sed -i -e "s/MEAN_DEPTH/M_DEPTH_$n/" $f;
 done
```

6.1.Run the script “sexing.R” inside the “sexing” directory to calculate Index_X and Index_Y and to reveal the sex of each individual.

```
Rscript sexing.R
```

Two new files will appear when it finishes, “final_sexing.csv”, containing results and “sexing_plots.pdf”, containing the different plots. Next, there are examples of the output files.

First, an example of the table “final_sexing.csv”.

	Sample type	Mean_cover	M_DEPTH_A	F_MISS_A	M_DEPTH_X	F_MISS_X	F_MISS_Y	Index_X	Index_Y	Sex
BEC12	Tissue (live a	22.846	11.6127	0.568431	8.26186	0.794881	0.526316	0.7114504	-1.3093131	male
BEC18	Fresh tissue (	23.6	20.3945	0.0339341	13.6792	0.0802091	0.116959	0.67072985	0.03995462	male
BEC20	Fresh tissue (	19.158	5.95634	0.753309	4.89126	0.907174	0.660819	0.82118549	-2.6539439	male
CO16	Fresh tissue (	24.959	22.1006	0.0347085	21.6207	0.0228911	0.947368	0.97828566	0.94613497	female
CO19	Fresh tissue (	21.649	28.1388	0.0185159	26.5133	0.0153208	0.947368	0.94223279	0.94654909	female
CO20	Fresh tissue (	4.564	30.0774	0.0837088	17.7779	0.225847	0.122807	0.5910717	-0.1331003	male
IB05	Fresh tissue (	17.908	27.7358	0.0720924	25.8482	0.146539	0.959064	0.93194355	0.9520353	female

And second, an example of the figures.

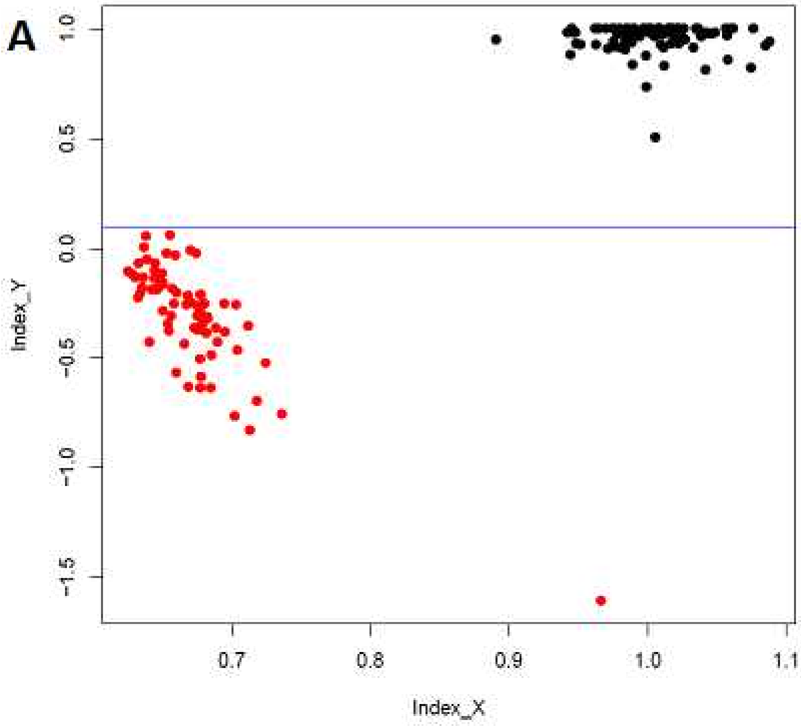

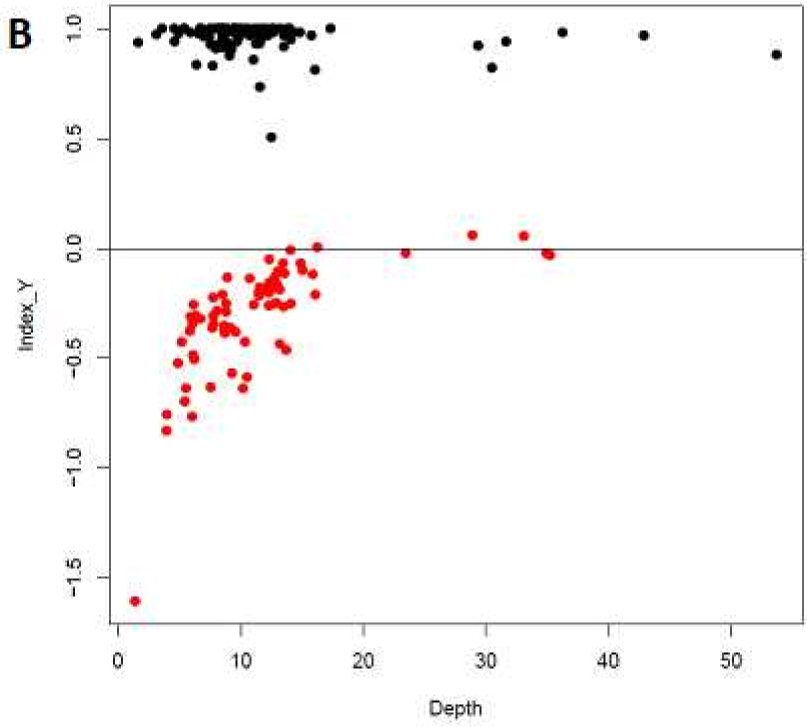

Figure A corresponds to a dispersion plot of Index Y and X. Figure B corresponds to one of the control figures showing Index Y vs Coverage depth. In both cases, red dots include male individuals, and black dots include females.

sexing.

5 setwd("./")

```
rm(list = ls())

list.files()

files_ind <- list.files(pattern="2.tsv")

##data frame creation

for (i in 1:length(files_ind)) assign(files_ind[i], read.table(files_ind[i],row.names =

1,check.names = F,header = T))

dfs <- Filter(function(x) is(x, "data.frame"), mget(ls()))
```

```
data_table <- merge(dfs[1],dfs[2],by =0,all =T)

rownames(data_table) <- data_table$Row.names

data_table$Row.names <- NULL


data_table <-merge(data_table, dfs[3],by =0, all= T)

rownames(data_table) <- data_table$Row.names

data_table$Row.names <- NULL


for (i in 4:length(dfs))

{

data_table <-merge(data_table, dfs[[i]],by =0, all= T)

rownames(data_table) <- data_table$Row.names

data_table$Row.names <- NULL

}


data_table[,4] <- NULL


colnames(data_table) <- c('M_DEPTH_A','M_DEPTH_X','F_MISS_X','F_MISS_Y')


##computing of X and Y indexes.


index_x <- data_table[2]/data_table[,1]

colnames(index_x)[1] <- "Index_X"


index_y <- ((1-data_table[3])-(1-data_table[,4]))/(1-data_table[,3])

colnames(index_y)[1] <- "Index_Y"
```

```
data_table_2 <- merge(data_table, index_x, by=0, all=TRUE)

rownames(data_table_2) <- data_table_2[,1]

data_table_2[,1] <- NULL


data_table_2 <- merge(data_table_2, index_y, by=0, all=TRUE)

rownames(data_table_2) <- data_table_2[,1]

data_table_2[,1] <- NULL


data_table_2$Sex[data_table_2$Index_Y <= 0.1 ] <- “male”

data_table_2$Sex[data_table_2$Index_Y > 0.1 ] <- “female”


##ploting


pdf(“sexing_plots.pdf”)

plot(x=data_table_2$Index_X, y=data_table_2$Index_Y, col=factor(data_table_2$Sex), xlab=“Index_X”, ylab=“Index_Y”, pch=19)

abline(h=0.1, col=“blue”)


plot(x=data_table_2$M_DEPTH_A, y=data_table_2$Index_X, col=factor(data_table_2$Sex), xlab=“Depth”, ylab=“Index_X”, pch=19)

abline(h=1, col=“black”)


plot(x=data_table_2$M_DEPTH_A, y=data_table_2$Index_Y, col=factor(data_table_2$Sex), xlab=“Depth”, ylab=“Index_Y”, pch=19)

abline(h=0, col=“black”)

dev.off()
```

```
write.csv (data_table_2, “final_sexing.csv”)
```
