# Supplementary material for: A rapid approach for sex assignment by RAD-seq using a reference genome
Source: PLoS One. 2024 Apr 5;19(4):e0297987. doi: 10.1371/journal.pone.0297987 (PMC10997085; doi:10.1371/journal.pone.0297987)

Figure S1. Dispersion plots of Index X (A) and Index Y (B) vs Coverage depth for the fur seals (*Arctocephalus forsteri*) dataset from Stovall et al. (2018). Red dots correspond to males. Black dots correspond to females.

B

A


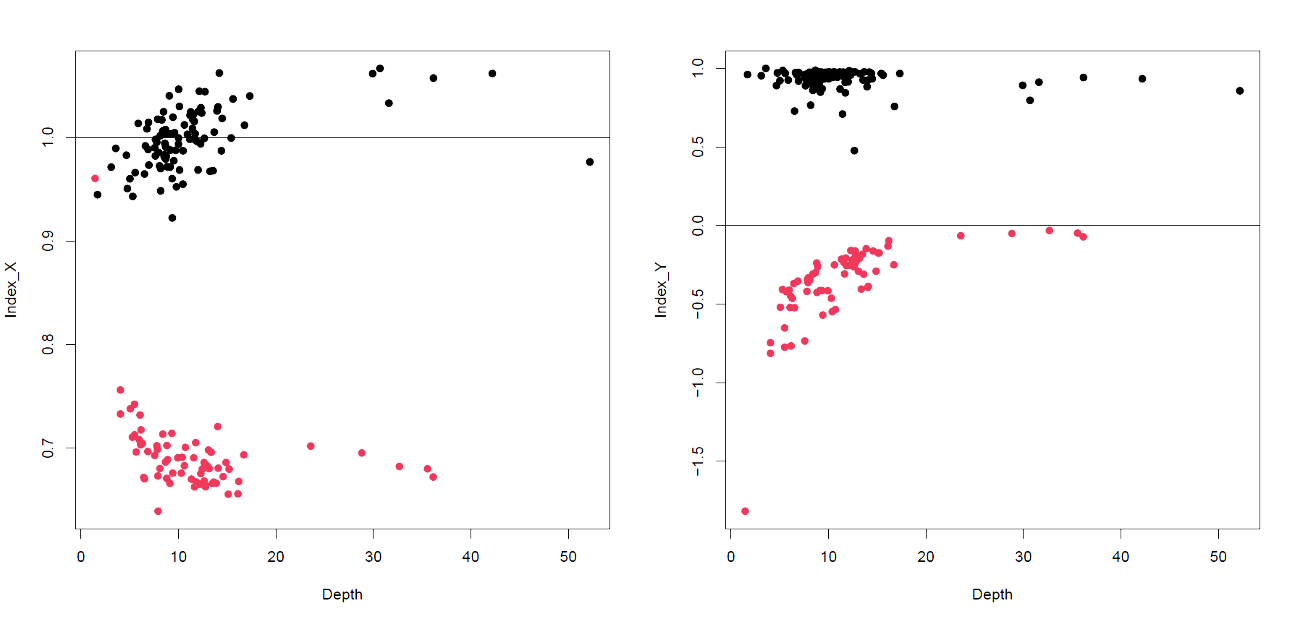


Figure S2. Dispersion plots of Index X (A) and Index Y (B) vs Coverage depth for the sea lions (*Otaria flavescens*) dataset. Red dots correspond to males. Black dots correspond to females.

B

A


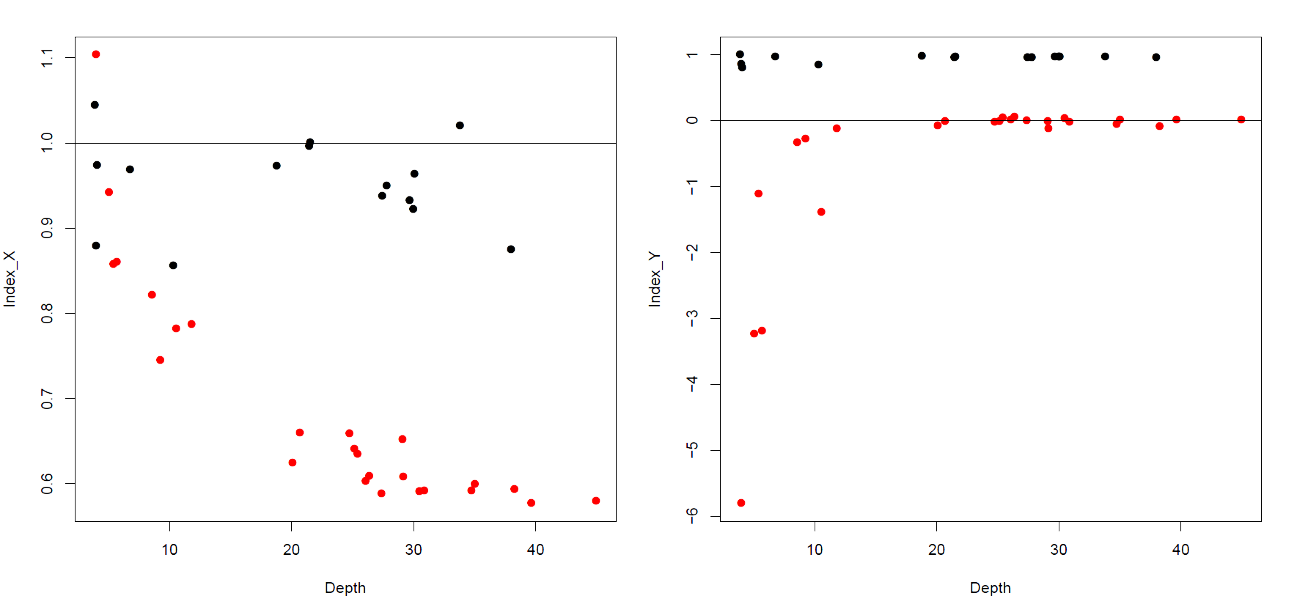

Supplement: S2 File — (DOCX) [file pone.0297987.s002.docx]
